# Supplementary material for: Genomic profiling of subcutaneous patient-derived xenografts reveals immune constraints on tumor evolution in childhood solid cancer
Source: Nat Commun. 2023 Nov 22;14:7600. doi: 10.1038/s41467-023-43373-1 (PMC10663468; doi:10.1038/s41467-023-43373-1)
Supplement: Supplementary file 1 — Supplementary Information [file 41467_2023_43373_MOESM1_ESM.pdf]

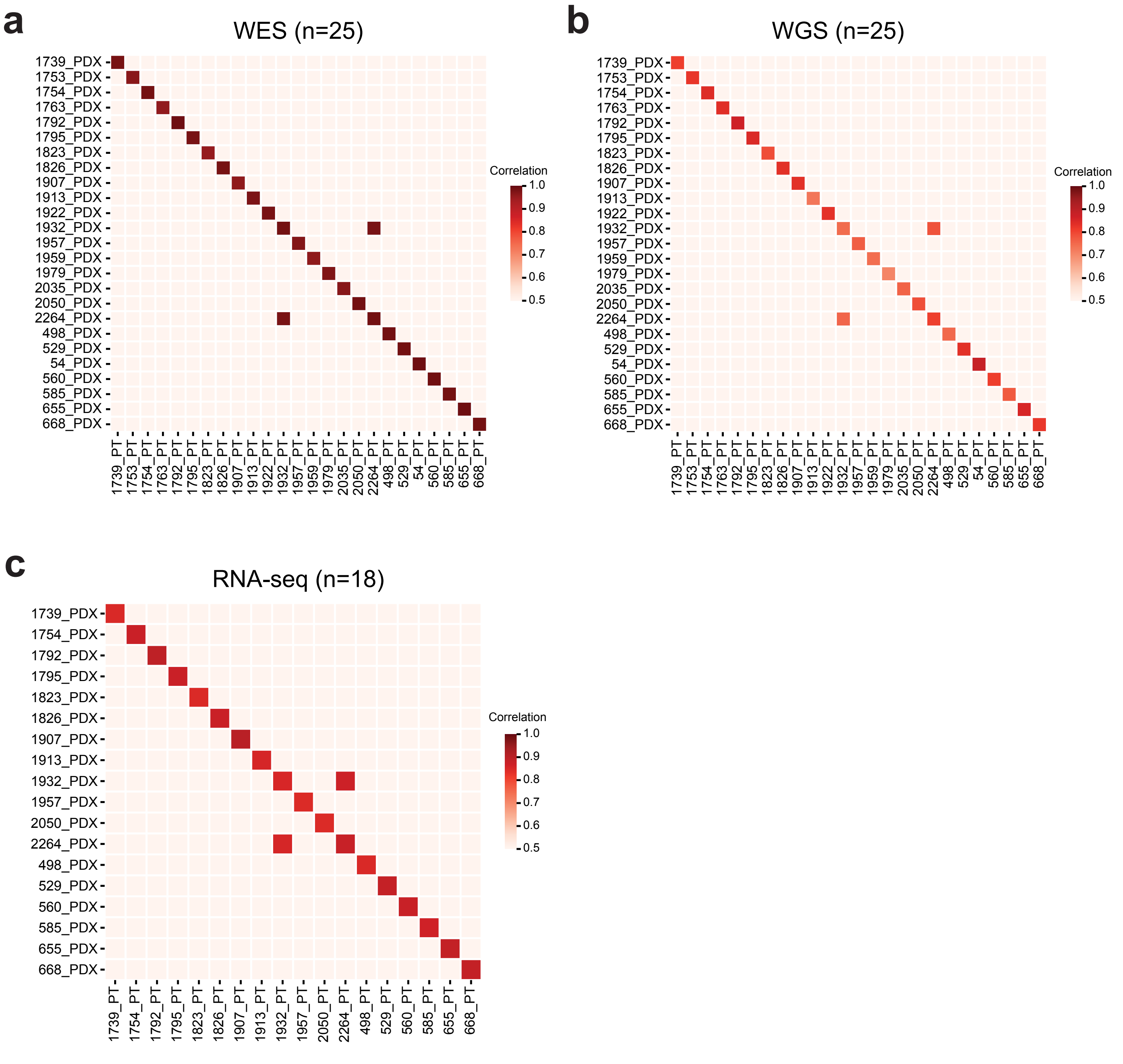

Supplementary Fig. 1 Genetic isogeneity based on ~20K SNPs between PTs and PDXs, using (**a**) whole exome sequencing data, (**b**) low pass whole genome sequencing data, and (**c**) RNA sequencing data. Samples 1932 and 2264 were sampled at two time points from the same patient. Correlation values were reported by NGSCheckMate (Lee et al. 2017).

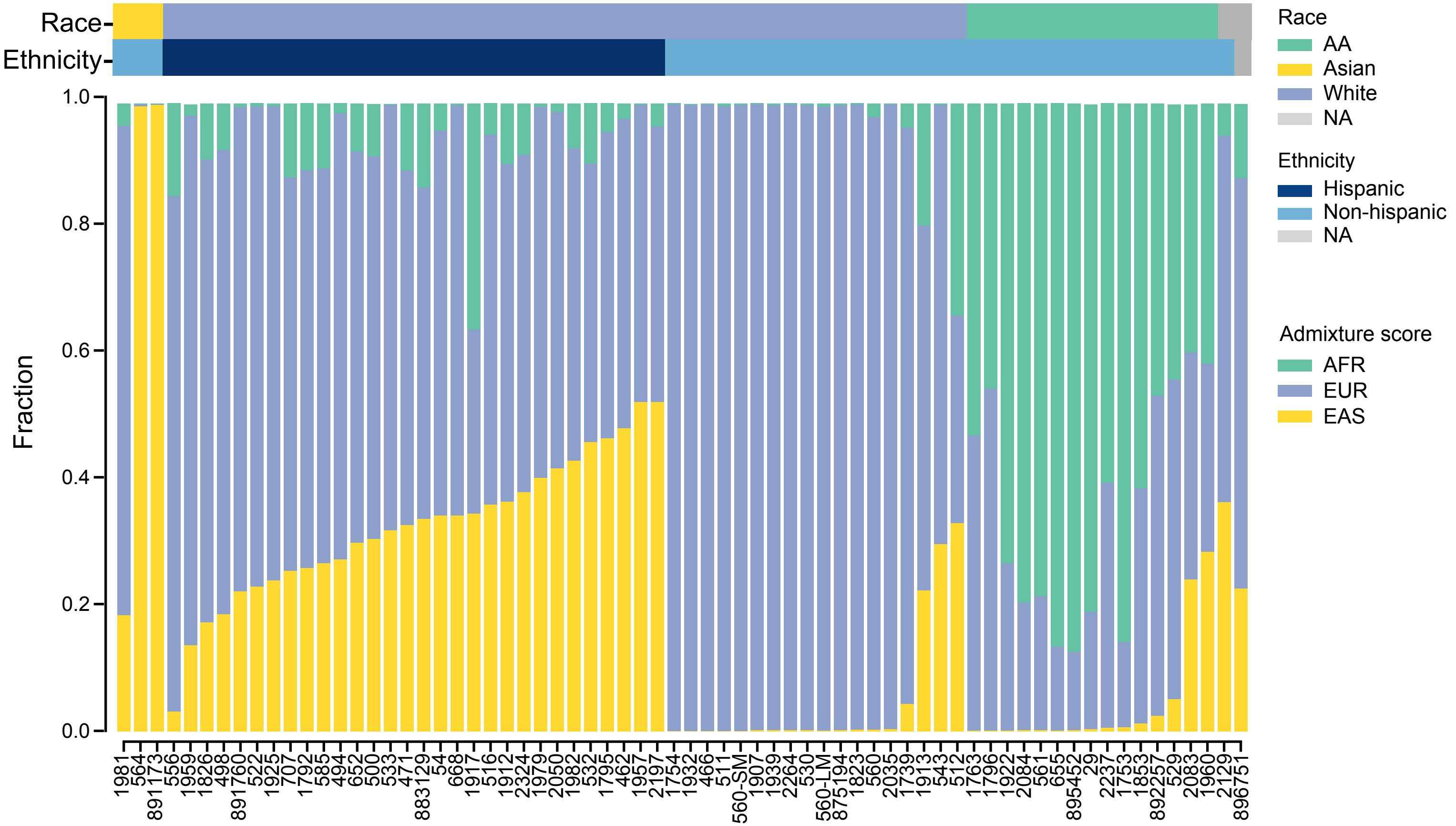

Supplementary Fig. 2 Confirmation of patient ancenstry. The top panel shows self-reported race and ethnicity. The bottom panel shows the admixture scores of African (AFR), European (EUR) and East Asian (EAS) populations. Self-reported hispanic patients show genetic mixture of AFR, EUR, and EAS.

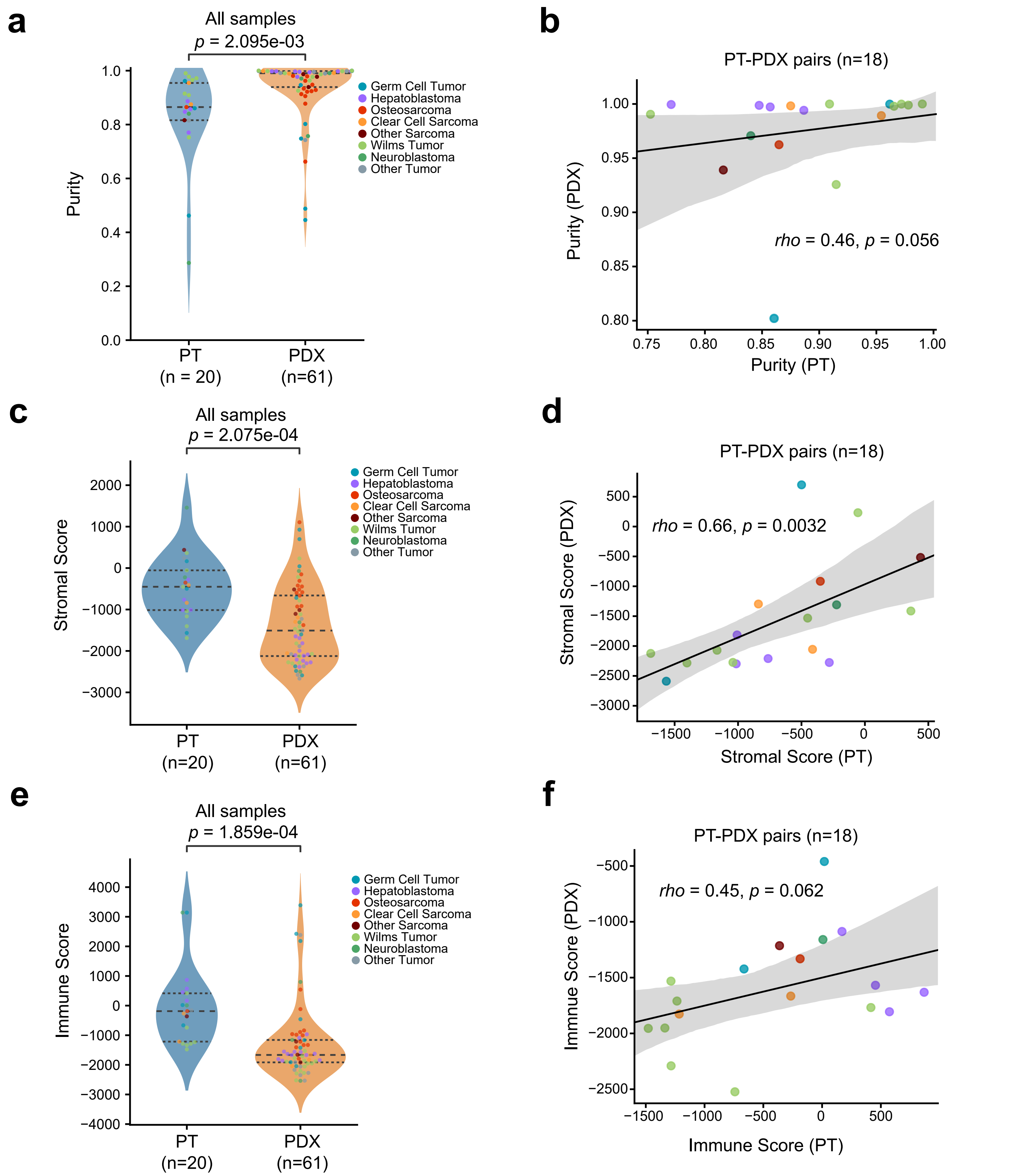

Supplementary Fig. 3 Tumor purity of PTs and PDXs. (**a**) Tumor purity of PT and PDX samples. (**b**) Spearman correlation of tumor purity in PT-PDX paired samples. (**c**) Stroma score of PT and PDX samples. (**d**) Spearman correlation of stromal scores in PT-PDX paired samples. (**e**) Immune score of PT and PDX samples. (**f**) Spearman correlation of immune score in PT-PDX paired samples. All these scores were calculated using ESTIMATE based on RNA-seq data. P-values were calculated with two-sided t-test. For violin plots, the center line represents the median, dashed lines represent the upper and lower quantiles. For scatter plots, solid black lines represent the linear regression line and shaded gray around the line represents 95% confidence interval.

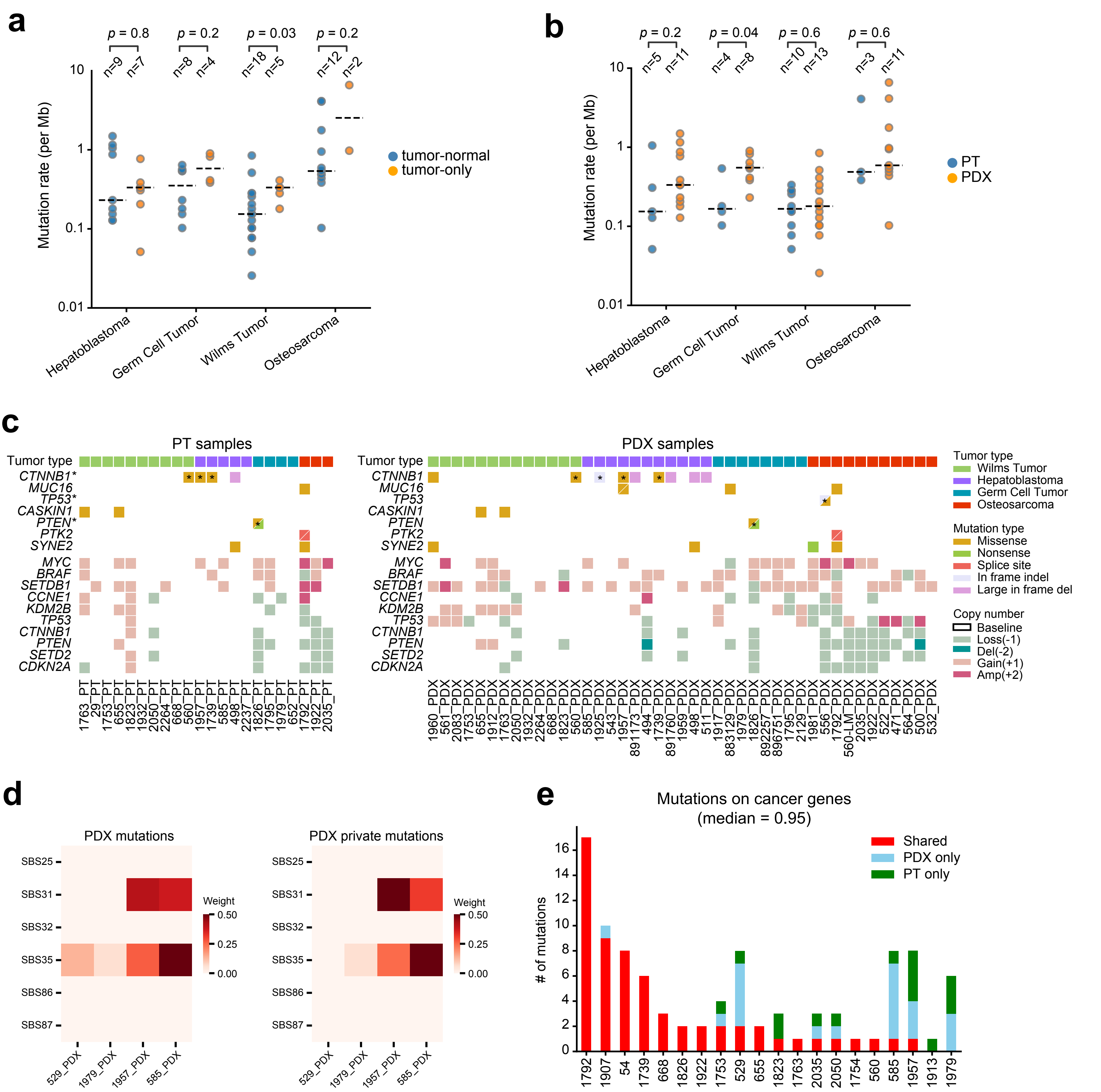

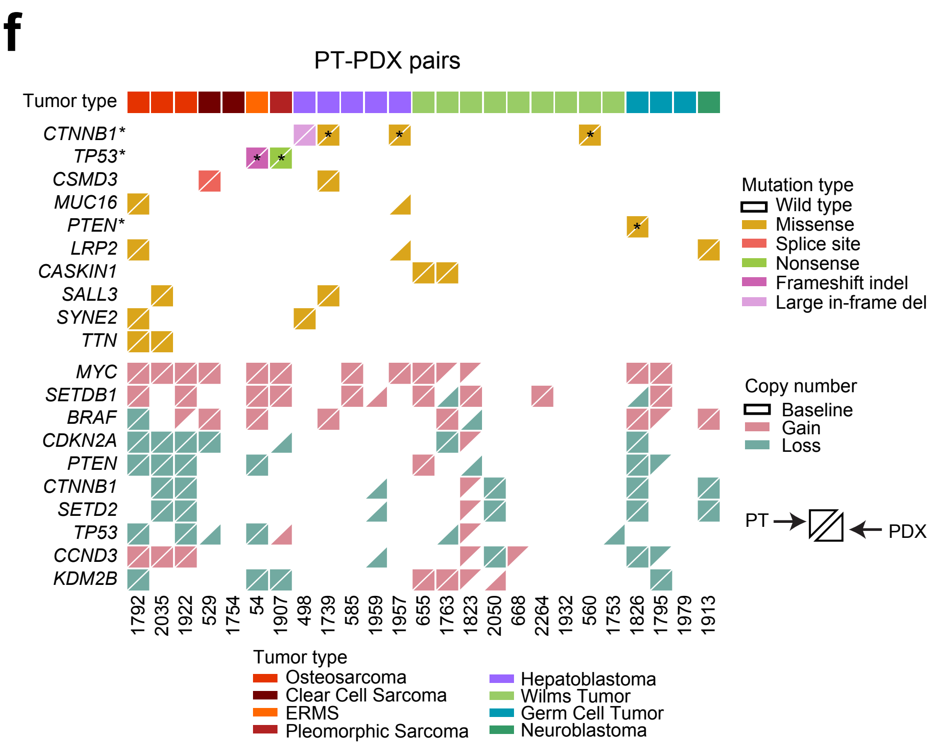

g

PT/PDX mutation similarity in multiPDX

	Shared	PT	PDX	Similarity
1795_PDX	5	7	9	0.45
1795-A_PDX	5	7	7	0.56
1795_PDX-2	5	7	7	0.56
1826_PDX	18	21	21	0.75
1826-A_PDX	20	21	26	0.74
1913_PDX	8	19	15	0.31
1913-A_PDX	8	19	23	0.24
1913-B_PDX	9	19	20	0.30
1932_PDX	4	6	5	0.57
1932-A_PDX	4	6	8	0.40
1932-B_PDX	4	6	7	0.44

Supplementary Fig. 4 Mutational similarity between PTs and PDXs. (**a**) Comparison of mutation rate between tumors with and without a matched normal. (**b**) Comparison of mutation rate between PT and PDX samples. Tumor subtypes with less than10 samples were dropped in comparisons. The p-values were calculated with two-sided Wilcoxon rank sum test. (**c**) Heatmap of cancer gene alterations in PTs and PDXs. Asterisks indicate oncogenic or likely oncogenic mutations classified by OncoKB-annotator. (**d**) Mutational signatures using all PDX mutations (left) or PDX specific mutations (right). (**e**) Mutation distance based on cancer genes. (**f**) Heatmap of cancer gene alterations in paired PT/PDXs. Upper half of each cell denotes PT, and the lower half denotes PDX. Asterisks indicate oncogenic or likely oncogenic mutations classified by OncoKB-annotator. (**g**) Mutation load in samples with multiple PDXs. For each pair, the first PDX listed in the table is the original PDX used in data analysis. PDXs designated as A or B were established from distinct patient tumor tissue in different mice. 1795_PDX-2 is a second tumor block of the original PDX. Numbers in the PT and PDX columns are the total number of mtations found in the sample.

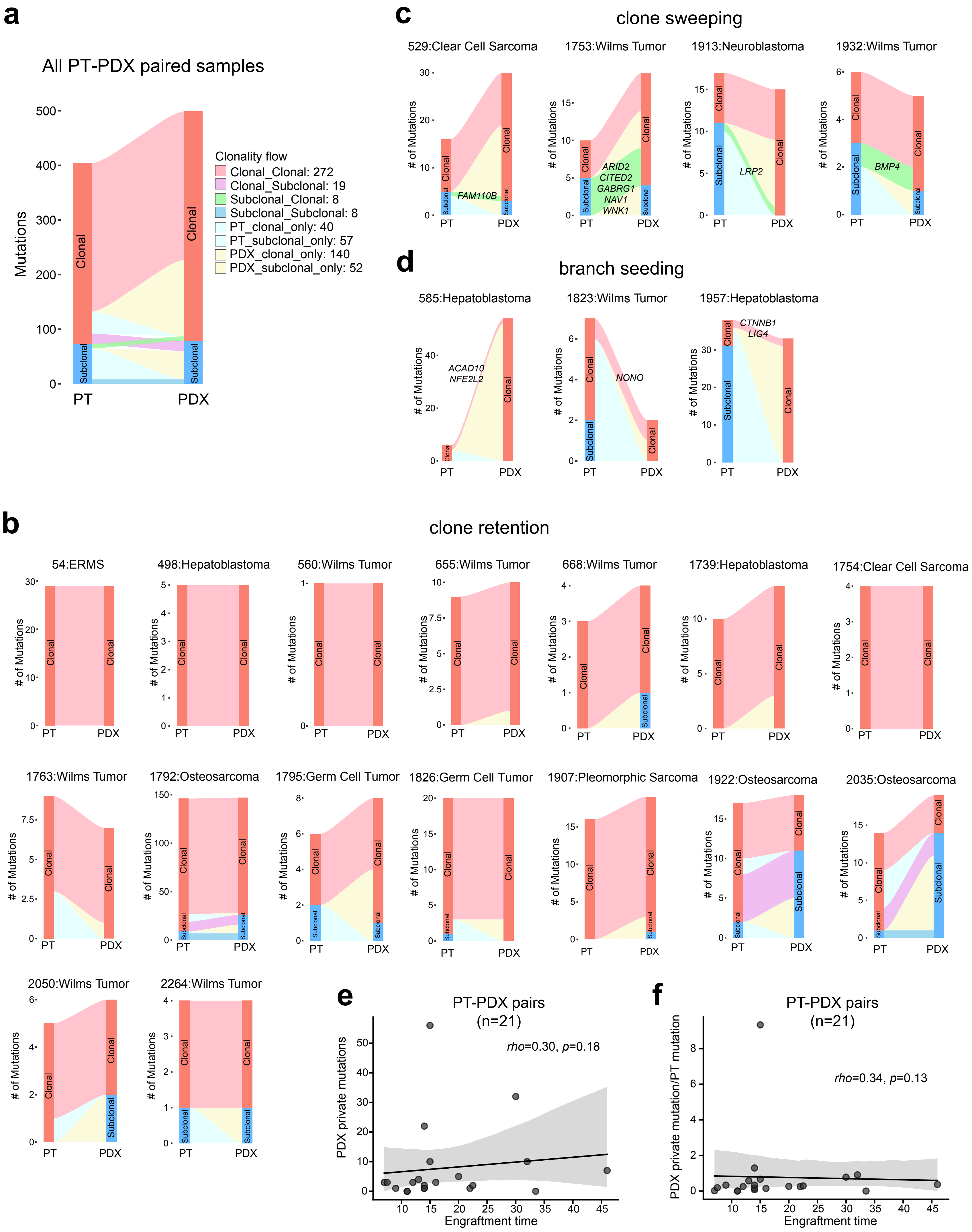

Supplementary Fig. 5 Evolution dynamic from PT to PDX. (**a**) Sankey plot of mutation clonality flow from PT to PDX for all paired PTs and PDXs. Red, clonal mutations; blue, subclonal mutations. Width of joining lines reflects the number of mutations. (**b**) Sankey plots showing clone retention cases, (**c**) clone sweeping, (**d**) branch seeding. (**e**) The number of PDX specific mutations is not correlated with engraftment time. (**f**) The same as (e), after controlling for PT mutation rates for each PDX. In e and f, the black solid line is the linear regression line, and the error band corresponds to 95% confidence interval.

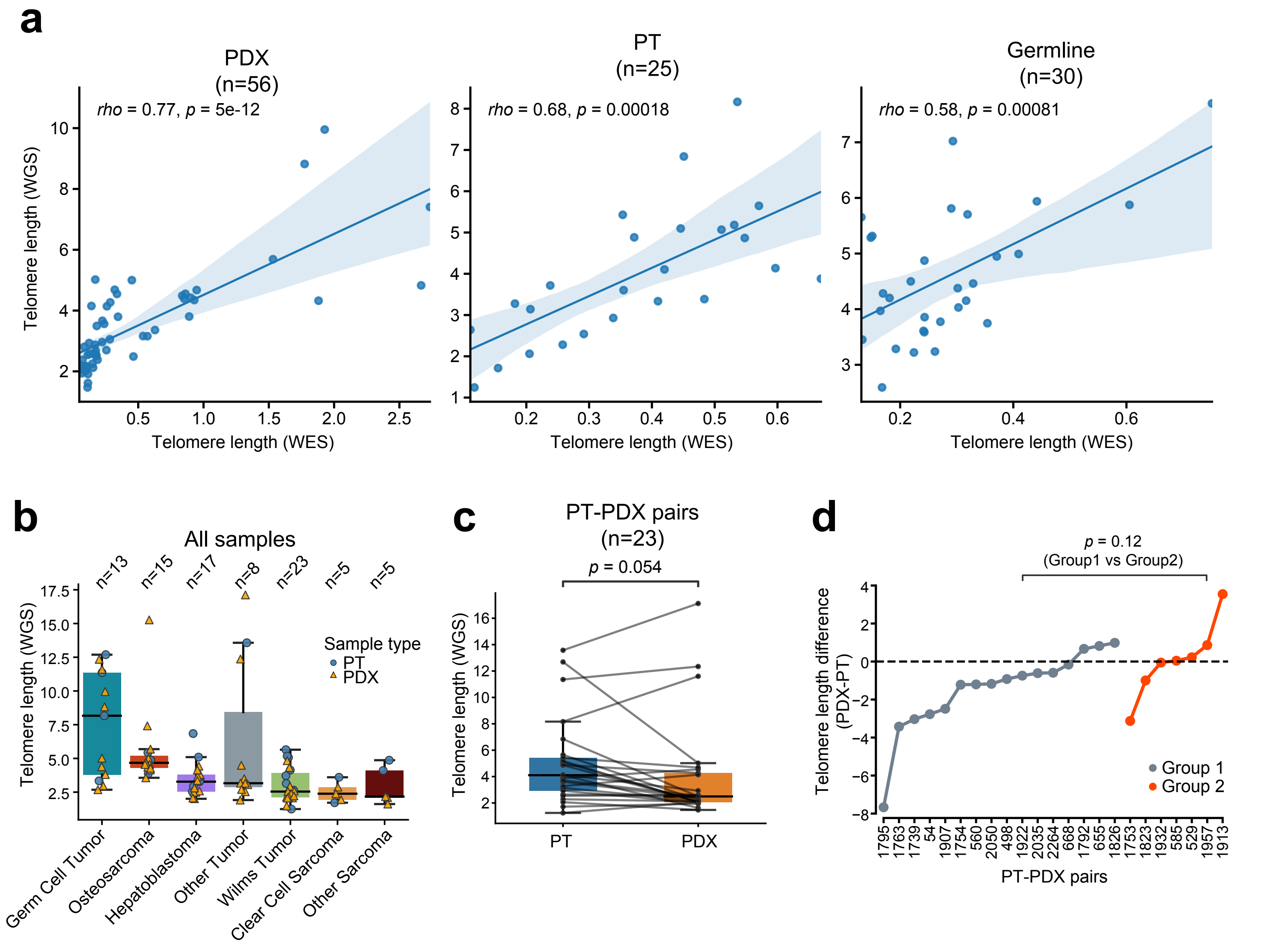

Supplementary Fig. 6 Telomere lengths of childhood cancer. (**a**) Spearman correlation of telomere length estimates from WES and WGS data. Telomere length was estimated usingTelseq. Each dot represents a sample. The solid blue lines represent the linear regression line and shaded blue around the line represents 95% confidence interval. (**b**) Distribution of telomere length based on WGS across tumor types. The boxes and middle lines within depict the interquartile range (IQR) and median. The top and bottom whiskers show values within 1.5 × IQR of the upper and lower quantiles respectively. (**c**) Differences in WGS-based telomere length estimates between PTs and PDXs. The lines connect matched samples. P value was calculated with two-sided paired t test. Boxplots are interpreted as above. (**d**) Differences in WGS-based telomere length estimates across the three evolutionary patterns. P value was calculated with two-sided Wilcox rank sum test.

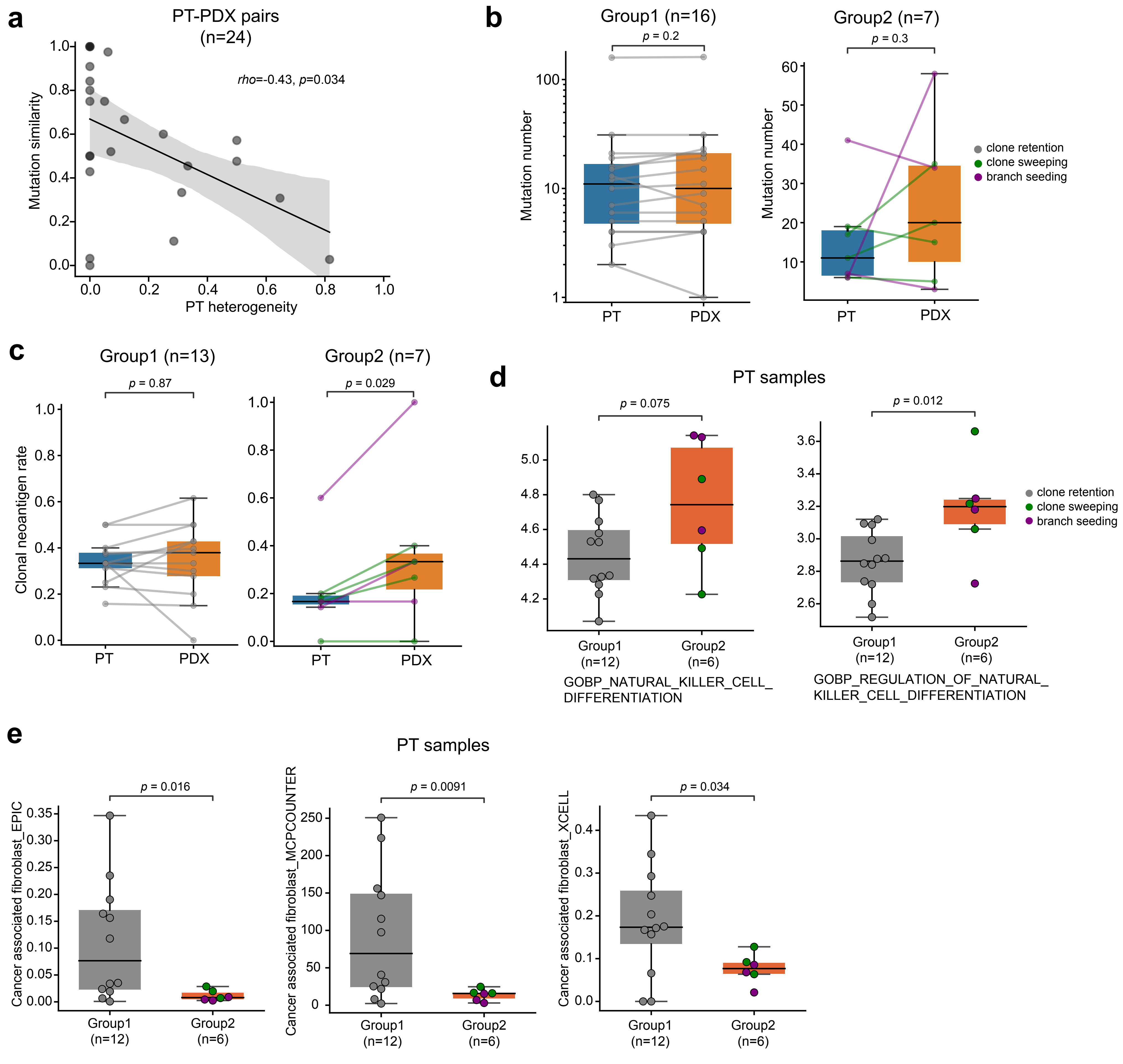

Supplementary Fig. 7 Comparison of tumor immunity. (**a**) The spearman correlation between mutation similarity and PT heterogeneity. PT heterogeneity was calculated by the fraction of subclonal mutations in PT. Each dot represents one PT/PDX pair. The black line is the linear regression line. Error band corresponds to 95% confidence interval. (**b**) Total mutational load in PTs and PDXs. Left, group 1 tumors; right, group 2 tumors. P value was calculated with two-sided paired t test. The box represents the interquartile range (IQR), and middle line represents median. Whiskers represent values within 1.5×IQR of the upper and lower quantiles respectively. (**c**) Comparison of clonal neoantigen rates (fraction of neoantigens over all clonal mutations). Left, group 1; Right, group 2. P value was calculated with two-sided paired t test. Boxplots are interpreted the same as above. (**d**) Expression of Natural Killer cell related signatures are higher in group 2 PTs. P values are calculated with one-sided Wilcoxon Rank Sum Test. Y axis is signature score calculated using ssGSEA. Boxplots are interpreted the same as above. (**e**) Abundance of cancer associated fibroblasts is lower in group 2 PTs. The three plots are based on outputs from EPIC (left), MCPCOUNTER (middle), and XCELL (right). These scores were calculated using the TIMER web service. P values were calculated with one-sided Wilcoxon rank sum test. Boxplots are interpreted the same as above.

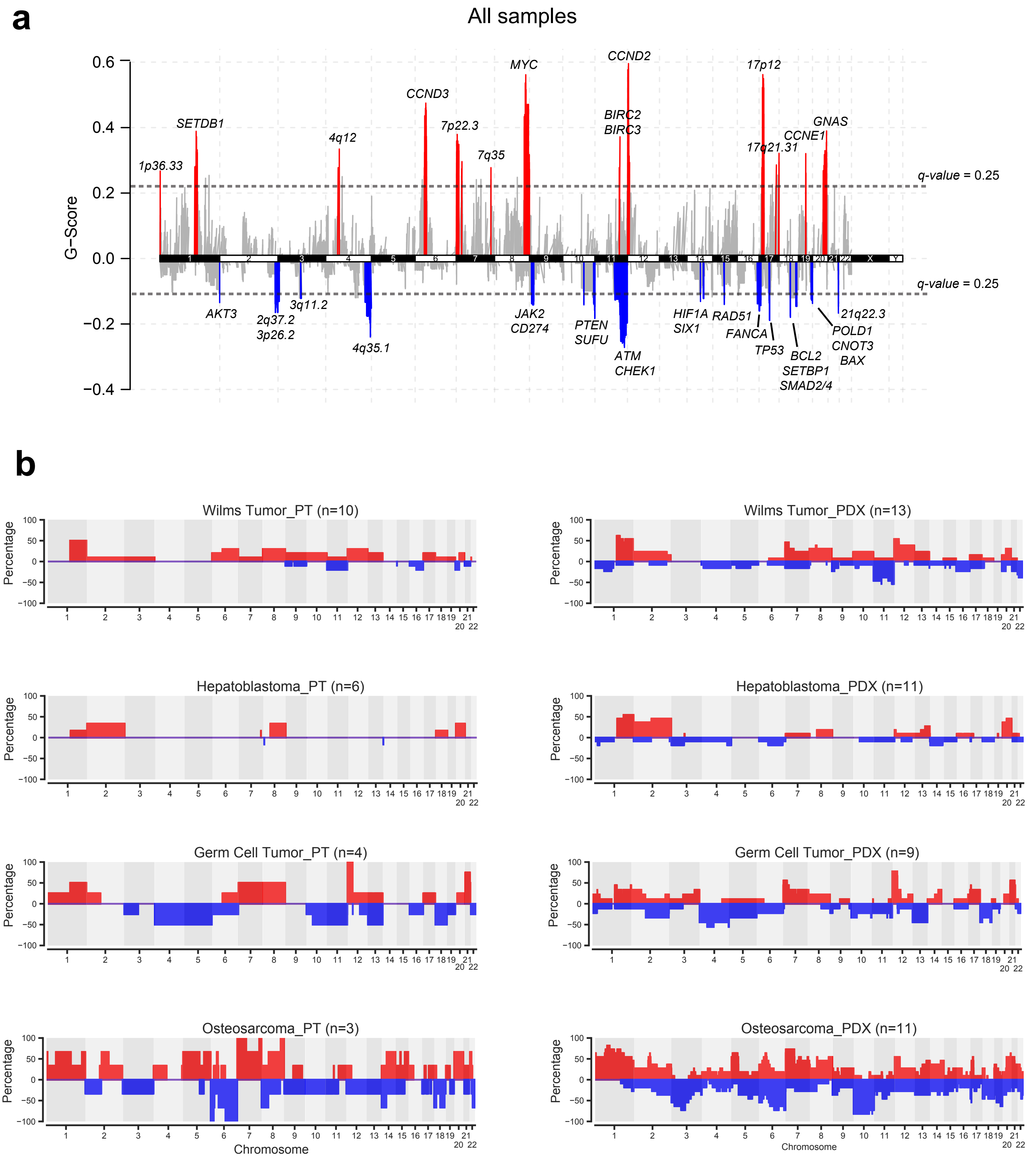

Supplementary Fig. 8 Copy number landscape of PDXs and PTs. (a) Recurrent amplification (red) and deletion peaks (blue). Highlighted are those with FDR<0.25. Cancer genes are noted for each peak. The peaks are called using GISTIC2. The y-axis represents GISTIC score. **(b)** Comparison of aggregated copy number changes from IGV between PTs and PDXs by cancer type. The y-axis in each plot represents the percentage of samples. Red, amplification; Blue, deletion.

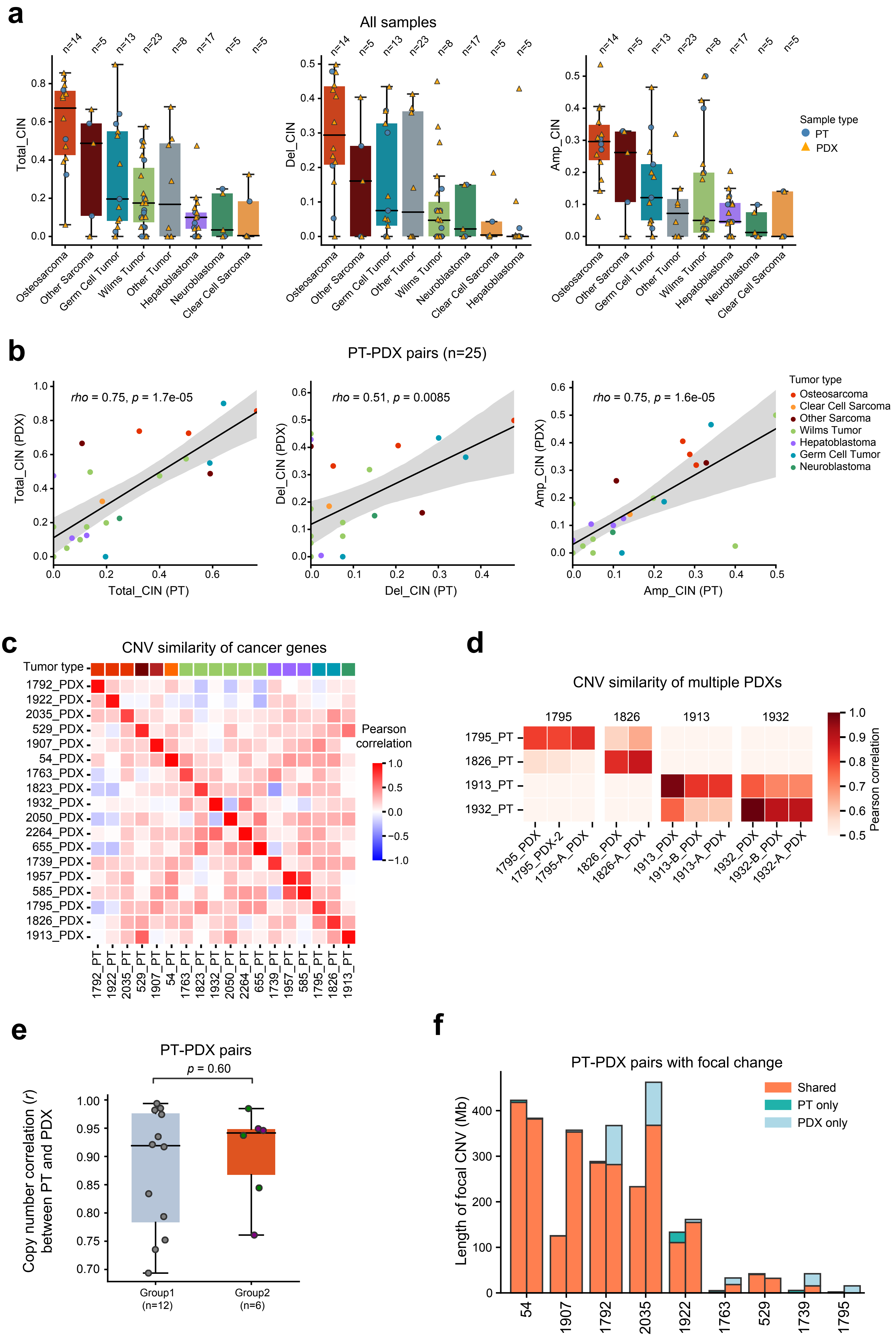

Supplementary Fig. 9 SCNA conservation in PDX. (**a**) Chromosome instability index (CIN) across pan-cancer, including total CIN (left), deletion CIN (middle) and amplification CIN (right). The box represents the interquartile range (IQR), and middle line represents median. Whiskers represent values within 1.5×IQR of the upper and lower quantiles respectively. (**b**) Spearman correlation of CIN in paired samples. The black line is the linear regression line. The error band represents 95% confidence interval. (**c**) Pairwise correlation of copy number profiles based on cancer genes. Samples with total CIN < 0.1 were excluded from the heatmap. (**d**). Pairwise correlation of copy number profiles in samples with multiple PDXs. (**e**) Correlation of copy number profiles across the three evolutionary patterns. P value was calculated with two-sided Wilcoxon rank sum test. Boxplots are interpreted as in a. (**f**) Length of focal events. The stacked bars represent length of focal CNV found in both PT and PDX (orange), PT only (green) and PDX only (blue). Bars on the left represent PTs, bars on the right represent PDXs.

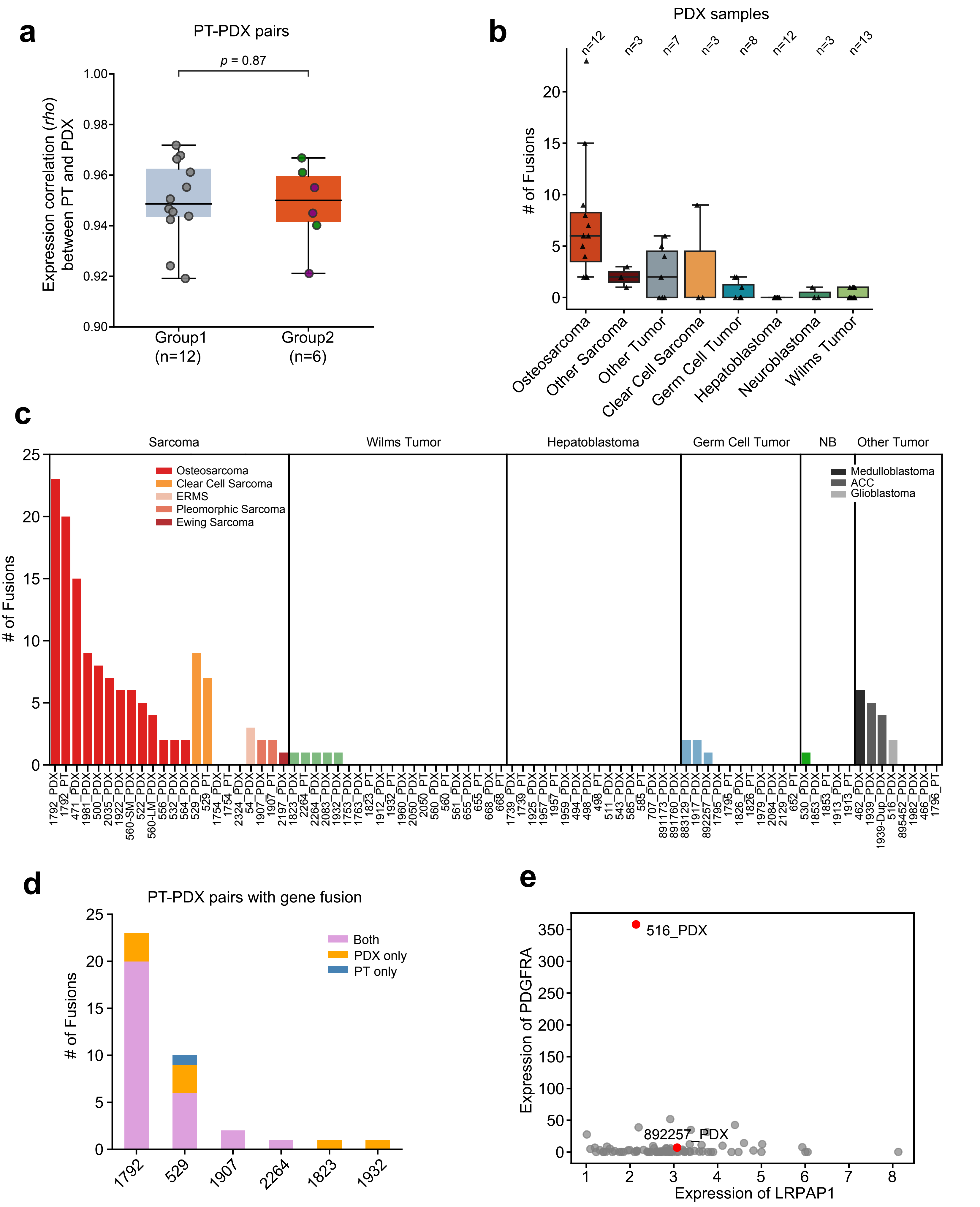

Supplementary Fig. 10 Expression conservation and gene fusion. (**a**) Pairwise correlation of gene expression between group 1 and group 2 tumors. The box represents the interquartile range (IQR), and middle line represents median. Whiskers represent values within 1.5×IQR of the upper and lower quantiles respectively. P value was calculated with two-sided Wilcoxon rank sum test. (**b**) Counts of gene fusions across tumor types. Each triangle represents a PDX sample. Boxplot is defined as in a. (**c**) Counts of Gene fusion per sample. (**d**) Overlap of gene fusions in six PT-PDX pairs. (**e**) The scatter plot of LRPAP1 and PDGFRA expression. Red dots represent samples with the LRPAP1-PDGFRA fusion.
