# Supplementary material for: Genomic profiling of subcutaneous patient-derived xenografts reveals immune constraints on tumor evolution in childhood solid cancer
Source: Nat Commun. 2023 Nov 22;14:7600. doi: 10.1038/s41467-023-43373-1 (PMC10663468; doi:10.1038/s41467-023-43373-1)
Supplement: Supplementary file 3 — Description of Additional Supplementary Files [file 41467_2023_43373_MOESM3_ESM.pdf]

Description of Additional Supplementary Files

Supplementary Datasets

Supplementary Data 1-

File Name: Supplementary Data 1.xlsx

Description: Patient clinical and demographic information

Supplementary Data 2-

File Name: Supplementary Data 2.xlsx

Description: Validation of somatic mutations using deep sequencing

Supplementary Data 3-

File Name: Supplementary Data 3.xlsx

Description: Mutation rates of driver genes in our PDX cohort and several other published cohorts.

Supplementary Data 4-

File Name: Supplementary Data 4.xlsx

Description: MutSigCV output on significantly mutated genes

Supplementary Data 5-

File Name: Supplementary Data 5.xlsx

Description: List of cancer genes used in PT/PDX genetic comparisons

Supplementary Data 6-

File Name: Supplementary Data 6.xlsx

Description: Telomere length estimates from whole genome and exome sequencing data

Supplementary Data 7-

File Name: Supplementary Data 7.xlsx

Description: Summary of PT/PDX mutation similarity and clonal neoantigens

Supplementary Data 8-

File Name: Supplementary Data 8.xlsx

Description: Top 20 pathways identified by GSEA between group 1 and group 2 PTs

Supplementary Data 9-

File Name: Supplementary Data 9.xlsx

Description: Detailed chromosomal instability scores of PTs and PDXs

Supplementary Data 10-

File Name: Supplementary Data 10.xlsx

Description: Summary of gene fusions identified in our cohort
