# Supplementary figures and images for: Genomic profiling of subcutaneous patient-derived xenografts reveals immune constraints on tumor evolution in childhood solid cancer
Source: Nat Commun. 2023 Nov 22;14:7600. doi: 10.1038/s41467-023-43373-1 (PMC10663468; doi:10.1038/s41467-023-43373-1)

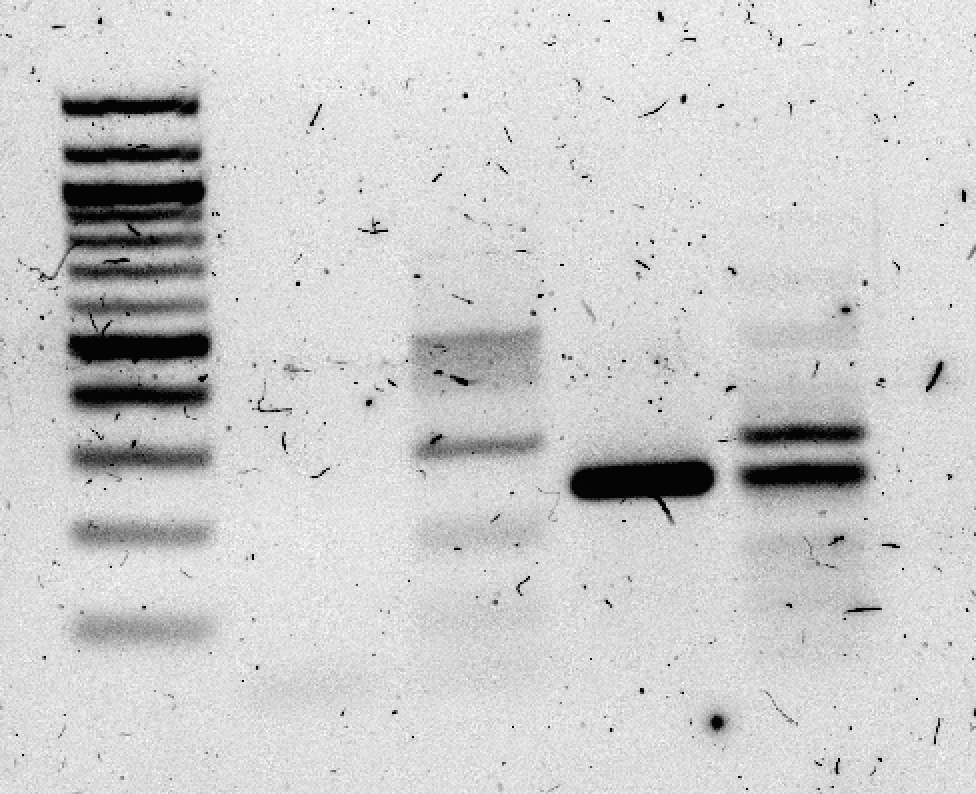

Supplement: Supplementary file 14 — Source data [file 41467_2023_43373_MOESM14_ESM.zip › Fig 6d exp 1.png]

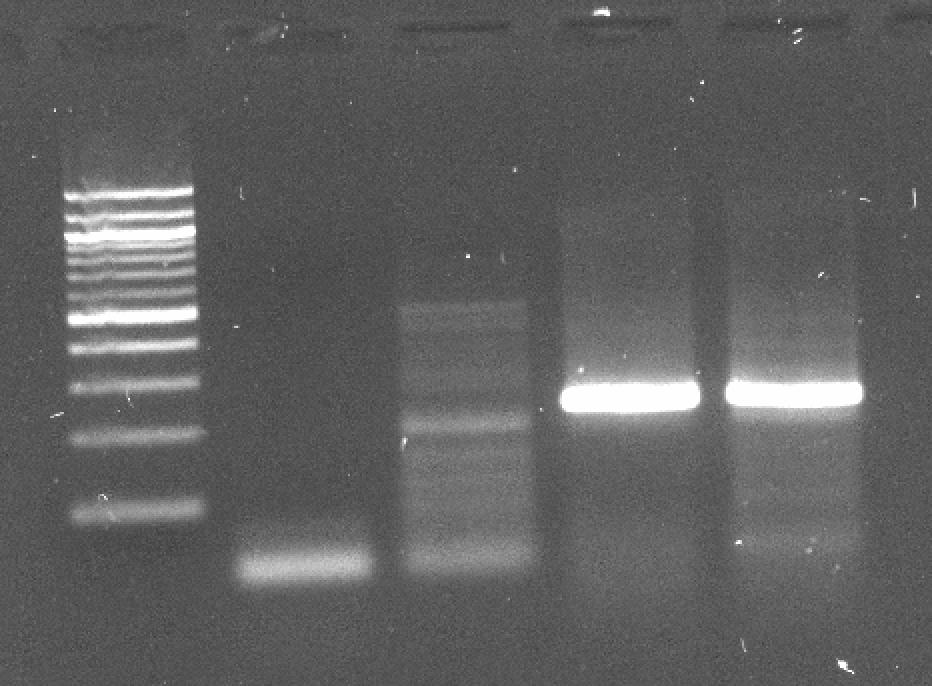

Supplement: Supplementary file 14 — Source data [file 41467_2023_43373_MOESM14_ESM.zip › Fig 6d exp 2.png]
